# Effectiveness of a therapeutic multiple-lifestyle intervention taking into account the periconceptional environment in the management of infertile couples: study design of a randomized controlled trial – the PEPCI study

**DOI:** 10.1186/s12884-020-2855-9

**Published:** 2020-05-26

**Authors:** Charlotte Dupont, Philippe Aegerter, Aude-Marie Foucaut, Aymeric Reyre, François J. Lhuissier, Marie Bourgain, Nathalie Chabbert-Buffet, Isabelle Cédrin-Durnerin, Lise Selleret, Emmanuel Cosson, Rachel Lévy

**Affiliations:** 1 Saint Antoine Research Center, INSERM équipe Lipodystrophies génétiques et acquises, Service de biologie de la reproduction-CECOS, AP-HP, Hôpital Tenon, Sorbonne Université, 4 rue de la Chine, F-75020 Paris, France; 2 Unité de Recherche Clinique et Département de Santé Publique, Assistance Publique - Hôpitaux de Paris, Hôpital Ambroise Paré, Boulogne, France; 3 UVSQ, UMR-S 1168 Université de Versailles Saint Quentin Paris Saclay, INSERM VIMA Aging and Chronic diseases, Epidemiological and Public Health approaches, Paris, France; 4 Université Sorbonne Paris Nord, Laboratoire Educations et Pratiques de Santé, LEPS, UR 3412, F-93017, Bobigny, France; 5 Département STAPS, Université Sorbonne Paris Nord, Bobigny, France; 6 Department of Addiction Medicine and Psychiatry, APHP Avicenne University Hospital, Bobigny, France; 7 CESP-INSERM U1178, Villejuif, France; 8 Université Sorbonne Paris Nord, UMR INSERM 1272 Hypoxie et poumon, Bobigny, France; 9 Assistance Publique - Hôpitaux de Paris, Hôpitaux Universitaires Paris Seine-Saint-Denis, Hôpital Jean Verdier, Médecine de l’exercice et du sport, Bondy, France; 10 Service de Gynécologie-Obstétrique et Médecine de La Reproduction, APHP Hôpital Tenon, 4 Rue de La Chine, 75020 Paris, France; 11 APHP Hôpital Jean verdier Service de médecine de la reproduction et préservation de la fertilité, avenue du 14-Juillet, 93143 Bondy, France; 12 Paris 13 University, Sorbonne Paris Cité, Assistance Publique - Hôpitaux de Paris, Avicenne Hospital, Department of Endocrinology-Diabetology-Nutrition, CRNH-IdF, CINFO, Bobigny, France; 13 Paris 13 University, Sorbonne Paris Cité, UMR U557 INSERM/U11125 INRA/CNAM/Université Paris13, Unité de Recherche Epidémiologique Nutritionnelle, Bobigny, France

**Keywords:** Fertility, Environment, Lifestyle, Periconceptional, Couple, Intervention

## Abstract

**Background:**

Infertility is defined as the inability to conceive after 12 months of unprotected intercourse. It affects approximately one in six couples seeking pregnancy in France or western countries. Many lifestyle factors of the couples’ pre and peri-conceptional environment (weight, diet, alcohol, tobacco, coffee, drugs, physical activity, stress, sleep…) have been identified as risk factors for infertility in both males and females. The high prevalence rates of unhealthy diets and lifestyles in the reproductive population of industrialized countries are worrisome. Nevertheless, adoption of a healthy lifestyle may improve fertility but lifestyle changes are difficult to achieve and to maintain due notably to behavioral factors.

**Methods:**

Consequently, we decided to propose an interventional study aimed at improving the quality of life of infertile couples before the start of assisted reproductive technology treatment. It is a randomized controlled multicentre trial. Both members of the couples are involved in an integrated global care program (PEPCI for “Parcours Environnement PériConceptionnel en Infertilité”) vs. usual care. This global intervention not only considers diet and/or physical activity but follows a holistic approach, including a multidisciplinary assessment to address complete physical, psychological and social well-being. According to patient needs, this includes interventions on weight, exercise, diet, alcohol and drugs, mental and social health.

**Discussion:**

The main objective of trial is to demonstrate that periconceptional multidisciplinary care has a positive impact on reproductive functions. We will also focus on feasibility, acceptance, compliance and conditions of success of a multifaceted lifestyle intervention.

**Trial registration:**

The trial was registered at ClinicalTrials.gov, Identifier: NCT02961907 on November 11, 2016.

## Background

### Lifestyle and infertility

Infertility is defined as the inability to conceive after 12 months of unprotected intercourse. It affects approximately one in six couples seeking pregnancy in France or other western countries [1]. Many lifestyle factors of the pre- and periconceptional environment (e.g., weight, diet, alcohol, tobacco, coffee, drugs, physical activity, stress, sleep) have been identified as risk factors for infertility in both males and females [2, 3]. Thus, the high prevalence rates of unhealthy diets and lifestyles in the reproductive population of industrialised countries are worrisome [4]. Although practitioners tend to focus more on the female environment, we believe it is also essential to consider the male environment.

#### Overweight, obesity, and metabolic syndrome

In women, it has been recognised for a long time that overweight, obesity, and metabolic syndrome have deleterious effects on fertility [5, 6]. A longer time to conceive and ovulation disorders are more often observed in overweight and obese women [7, 8]. In addition, an increased risk of miscarriages and a decrease in the chances of achieving pregnancy after assisted reproductive technology (ART) exposure have also been described [9]. A meta-analysis including 21 studies has highlighted that female obesity negatively impacts live birth rates following in vitro fertilisation with a risk ratio (RR) [(95% confidence interval (CI)] of 0.85 (0.82–0.87) [10].

Among men, existing data are more recent yet still scarce. A meta-analysis including 13,077 men concluded the deleterious impact of overweight and obesity on sperm production [11]. Obesity also appears to have a negative impact on sperm DNA integrity [12]. Furthermore, male overweight and obesity seem to be detrimental to attaining successful ART results [13, 14]. Lastly, in comparing 100 infertile couples and 100 fertile couples, we observed a link between metabolic syndrome and male idiopathic infertility [15].

#### Weight loss

Weight loss is encouraged in cases of overweight and obesity. In overweight or obese women, weight loss, even moderate, may facilitate cycle regularisation and a spontaneous recovery of ovulation [16]. Such also increases spontaneous pregnancies [17, 18]. Nevertheless, the impact of weight loss on the chances of success in ART is less obvious [19, 20]. Mutsaerts et al. recently reported that rates of ongoing pregnancy and clinical pregnancy were not different between obese infertile women who have followed an interventional program aiming to achieve weight loss and those receiving prompt infertility treatment [19]. The authors suggested that a more intensive program or one involving better strategies to enhance adherence might have resulted in a better rate of pregnancy.

Concerning trials focused on the reproductive functions of obese men, an improvement in hormonal balance and erectile function has been observed after weight loss [21]. However, the impact of weight loss on sperm parameters or on ART results has rarely been studied. Only two studies including 43 and 200 men, respectively, have observed an improvement in sperm parameters after weight loss [22, 23]. Elsewhere, one study highlighted an improvement in sperm DNA integrity after weight loss in 126 obese men [24].

#### Physical activity

Although limited, some observational studies have highlighted the importance of moderate physical activity for fertility in both males and females [25, 26]. Recently, in a case–control study involving 302 men and women, we observed that physical inactivity was correlated with infertility in men [odds ratio (OR): 2.20; 95% CI: 1.06–4.58]. In this study, sedentary behaviour was also linked to female infertility (OR: 3.61; 95% CI: 1.58–8.24) [27].

#### Diet quality pattern

Overweight and obesity are not the only factors to consider; adequate diet and an appropriate intake of recommended daily allowances are similarly crucial to the success of pregnancy [28, 29]. Indeed, several studies have shown the deleterious effect of certain eating behaviours on male and female reproductive functions; for example, an insufficient intake of vegetables and fruits, cereals with sufficient fibre, foods rich in omega 3 (e.g., fatty fish, avocados), poultry, foods rich in antioxidants, and low-fat dairy products increases the risk of infertility. In addition, high rates of consumption of high-fat dairy products (cheese), potatoes, soy-based foods, red or processed meat, saturated fatty acids and sugars, coffee, and alcohol are harmful [30–32].

#### Other lifestyle factors

Besides diet and physical activity, addiction (tobacco, alcohol, and drug use), stress, and sleep are other modifiable factors that should be considered. It is recognised that a high level of consumption of tobacco, alcohol, or caffeinated beverages can have a negative impact on both female and male fertility [3, 33–35]. Stress has been associated with a negative impact on male fertility (i.e., on sperm concentration, mobility, and morphology) [36]. In females, stress may increase the time to pregnancy (TTP) [37] or accelerate ovarian reserve exhaustion in young women [38]. Sleep disturbances have been associated with altered semen parameters [39]. In women, it was suggested that sleep disturbances (and inherent circadian rhythm) lead to menstrual irregularities [40] and are associated with reproductive dysfunction [41].

### Adoption of a healthy lifestyle and fertility

Consequently, adoption of a healthy lifestyle may improve fertility as observed by several research teams [16, 42–44] including ours [45]. However, lifestyle changes are difficult to achieve and to maintain due notably to behavioural factors.

A Cochrane review indicated that no randomised controlled trial had yet assessed the effects of preconception advice or interventions on the chance of a live birth or other fertility outcomes in humans. The authors highlighted the need for further research in this important field [46].

Furthermore, despite the available knowledge on the relationship between poor nutrition or unhealthy lifestyle and the risk of infertility, congenital malformations, and maternal complications, health professionals and parents-to-be often remain unaware of the adverse effects of such a lifestyle [47].

Another key point is the target: most, if not all trials, focus on women only, although (1), unhealthy behaviours negatively affect the reproductive function of both parents; (2) a child is a common project; and (3) emulation can help parents to support each other, while the inclusion of both parents, even the one without any obvious lifestyle problems, might be viewed as a less confronting and more acceptable approach [48, 49].

Lastly, because interventions cannot fit all populations in the same way, tailoring intervention content and offering personalised behavioural and action feedback might increase the effectiveness of the programs compared to generic or so-called ‘one-size-fits-all’ interventions [50]. However, busy work schedules and travel constraints may affect participation rates, so, relative to face-to-face interventions, video-conference counselling sessions delivered via the Internet are more easily accessible and cost-effective. Online communication favours the multiplication of assessment points over time that allow for dynamically tailored interventions, which are credited to be more effective [51]. Thus, ease of use and high personal relevance for participants may also limit dropouts during the study [52]. Indeed, previous studies on computer-tailored web-based interventions reported positive results for a variety of health behaviours [53].

The available evidence justifies a reorganisation of infertility care for those who intend to change their unhealthy lifestyles, which includes a global preconceptional evaluation and personalised face-to-face counselling [43, 54]. Thus, we have decided to propose an interventional study aimed at improving the quality of life of infertile couples before the start of ART treatment. Our global intervention not only considers diet and/or physical activity but follows a holistic approach, including a multidisciplinary assessment to address complete physical, psychological, and social well-being. According to patient needs, the intervention includes elements on weight, exercise, diet, alcohol and drugs, and mental and social health [55]. Unlike many studies that included only overweight or obese patients, we involve both members of the parents-to-be with a body mass index (BMI) of less than 49 kg/m^2^.

## Objectives and outcomes

The main objective of this randomised study is to demonstrate that periconceptional multidisciplinary care aiming to evaluate and optimise habits of infertile couples (i.e., diet and lifestyle factors) has a positive impact on reproductive functions as a whole, including birth issues, relative to a routine approach. We will also focus on feasibility, acceptance, compliance, and conditions of success of a multifaceted lifestyle intervention.

The primary outcome of this trial is the rate of clinical ongoing pregnancies (ultrasound determination of a gestational sac at 6 weeks of amenorrhea), either after the first ART attempt performed within three to 12 months of the initial visit or during a spontaneous pregnancy occurring within 12 months after the initial visit. Couples who do not conceive during the study period (within 12 months of the initial visit) will be considered as failures. Ultrasound performed at 6 weeks of amenorrhea will be performed by an operator blinded to the intervention arm.

The secondary outcomes will allow us to compare the two arms of the trial regarding (1) the couple’s adherence, satisfaction with the program, and understanding of the importance of receiving care; (2) changes in risk factors (e.g., nutrition, addiction, physical activity, psychological factors); (3) evolution of metabolic biomarkers (including folic acid); (5) sperm quality evolution (conventional semen parameters and DNA integrity); (5) TTP; and (6) quality of life. In cases of ART, we will assess outcomes including fertilisation rate, top quality embryo rate, implantation rate, and the number of ART procedure attempts needed to achieve pregnancy within 12 months.

The tertiary outcomes concern a subgroup of pregnant women followed until delivery, through evaluation of (1) maternal complications and pregnancy outcomes (e.g., prematurity, pregnancy living), (2) newborn check-up results, (3) quality of breast milk, and (4) further analyses of collected cord blood.

## Methods

### Experimental design

We propose a pragmatic randomised controlled multicentre trial of the improved effectiveness of an integrated global care program (PEPCI for Parcours Environnement PériConceptionnel en Infertilité) in comparison with usual care (i.e., a parallel-arm superiority trial). Four centres in Paris and its suburb area with various socioeconomic contexts will participate. Use of a pragmatic approach and an effectiveness assessment will assume not only clinical efficacy but also feasibility issues such as acceptability of care by patients under usual conditions as reflected by wide selection criteria [56]. Thus, this trial will use the ‘cohort multiple randomised controlled trial’ methodology [57] to strengthen external validity by limiting the bias that may result from the period of initial consent to randomisation or from the deception of being allocated to the control group.

Moreover, this design allows us to keep all people under survey to obtain realistic estimates of noncompliance and effectiveness of the program. It reproduces ‘patient-centred’ informed consent, in that the process of obtaining patient information and consent aims to replicate the one used in real-world routine health care.

### Study recruitment

**Fig. 1:**
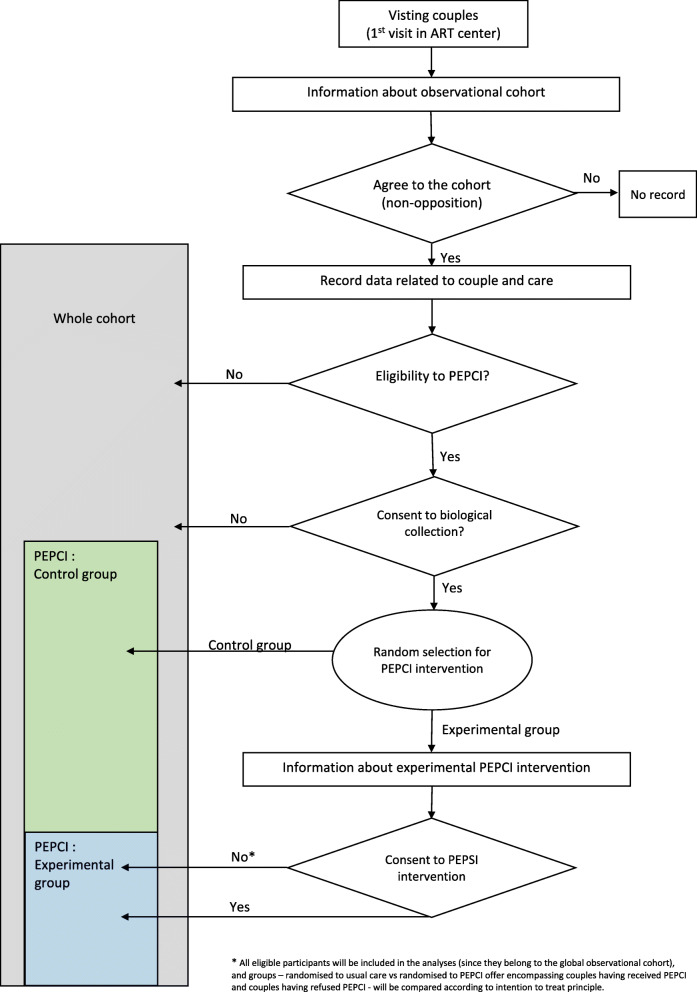
Recruitment and composition of the PEPCI study

Recruitment for the study will follow a three-step process (Fig. 1) as follows:

1. In practice, at their first visit to the ART centre, all the attending couples will be invited to participate in an observational survey cohort for evaluation purposes to facilitate our access to their data that are prospectively collected through standardised forms.
2. Unless a couple has refused to be included in the ‘whole’ cohort, they will be checked for eligibility as the consultation is going on and, if eligible, will be informed of such and asked for written consent for biological sample collection (biobank).
3. In the case of biobank consent, an ‘on-the-fly’ randomisation will be performed by the physician on a dedicated website that selects PEPCI candidate couples who are offered the PEPCI experimental intervention (experimental group). Thus, the process of obtaining the couple’s information and consent replicates the real-world routine health care and concerns the intervention only and not the trial. Couples randomised in the experimental program can refuse the experimental program. In this case, they will benefit from usual care. Couples randomised in the usual care group (control group) won’t need to provide any additional information since they already agreed to the collection of data and biological samples for evaluation purposes. The randomisation sequence is prepared in advance and stratified by centre with unrevealed varying block sizes, while allocation will be programmed according to a 2:1 control:experimental ratio in line with the sample size calculation and to minimise global cost.

Analyses will be performed according to an intention-to-treat principle and will compare, among eligible and biobank-agreeing patients, all randomised to usual care (control arm) participants to all candidate PEPCI participants, depending on whether they have consented to the PEPCI intervention or not. The trial will be conducted and reported according to the Consolidated Standards of Reporting Trials guidelines and their pragmatic extension [58].

### Selection criteria

#### Whole cohort

Heterosexual infertile couples (lack of pregnancy after 12 months of unprotected intercourse), including men aged 18 to 60 years and women aged 18 to 43 years will be eligible for inclusion in the whole cohort. Other main inclusion criteria include a good understanding of the French language by both couple members and the approval of data collection by both parents-to-be.

#### Eligible patients for the controlled trial

After inclusion in the study cohort, both members of the couple must give their informed written consent for the collection of their biological samples. In addition, according to French laws, they must be affiliated with a social security scheme (beneficiary or legal). Lastly, they must have access to an Internet-connected device with a webcam at home. They must not present any contraindication to adapted physical activity.

The main exclusion criteria are (1) gamete donation; (2) BMI of more than 40 kg/m^2^ for a member of the couple, requiring mandatory care; (3) viral context (i.e., one or both members of the couple shows human immunodeficiency virus, hepatitis B, C infection); (4) inability to comply with the care program—notably, the physical exercise regimen; (5) ongoing pregnancy; (6) medically-treated diabetes mellitus; and (7) psychiatric pathologies requiring or currently under treatment.

The different groups are shown in Fig. 1.

### Data collection procedures and assays

#### Whole cohort

All patients included in the whole cohort (including the PEPCI study) will undergo a baseline evaluation of diet and lifestyle factors through the following questionnaires:

- Dietary intake: ‘SUVIMAX questionnaire’ [59, 60] for 3 days, consecutive or not with a weekend day, including medication/dietary supplements use (multivitamins, folate) and binge-eating disorders (Binge-eating Scale) [61]
- Physical activity level: ‘International Physical Activity Questionnaire (IPAQ)’ [62, 63]
- Sleep quality: ‘Pittsburgh Sleep Quality Index (PSQI)’ [64, 65]
- Depression and anxiety troubles: ‘Hospital Anxiety and Depression (HAD) scale’ [66, 67]
- Quality of life: ‘Duke questionnaire’ [68]
- Locus of control and individual beliefs: French version of the C form of the Multidimensional Health Locus of Control [69] to understand factors of adherence to the program and pregnancy [52]

#### Patients involved in the controlled trial (Figs. 2 and 3)

All couples recruited in this trial will be assessed at baseline (M0), while the couples who accepted to undergo the PEPCI intervention will be evaluated 3 months later (M3). Then, all couples (control and experimental arms) will be followed for 12 months (M12) or evaluated at the time of pregnancy diagnosis and then until delivery.

**Fig. 2:**
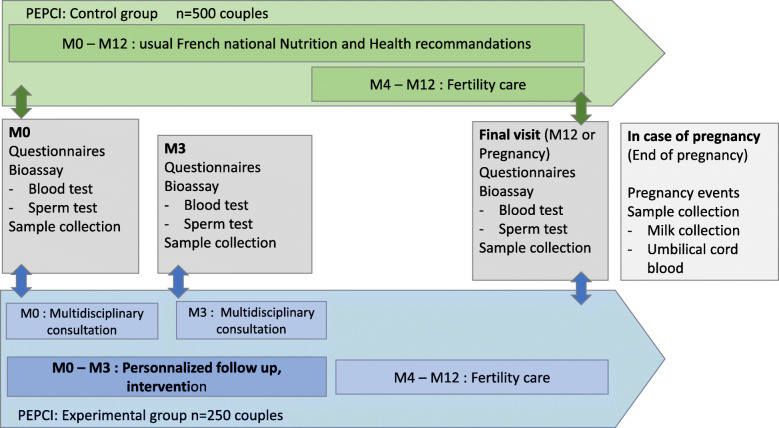
Progress of care during the PEPCI study. Green: control group; blue: experimental group

**Fig. 3:**
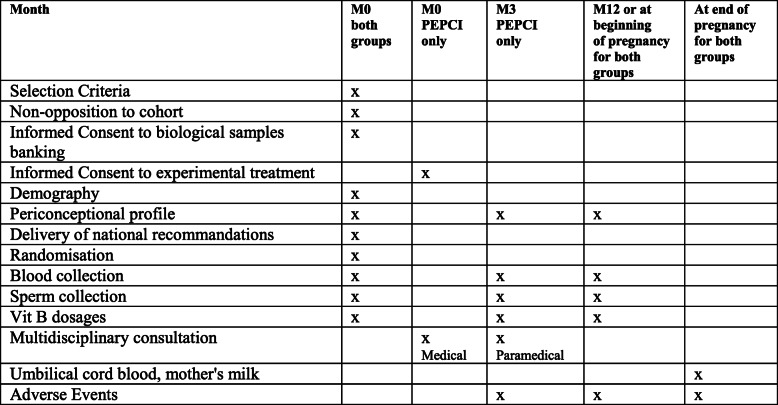
PEPCI study visits schedule

#### Data collection and assays at baseline (M0)

In both arms, blood (after a 12-h fasting period) and sperm will be collected for immediate and further analyses (biobank). Immediate analyses include (1) metabolic assays: measurement of total plasma cholesterol, high-density lipoprotein cholesterol (HDL), low-density lipoprotein cholesterol (LDL), triglycerides, and plasma glucose concentrations; (2) one-carbon metabolism assays: measurement of folate, homocysteine, and vitamins B6 and B12; and (3) sperm parameters assessment. Semen samples will be collected by masturbation in a sterile plastic cup after three to 5 days of sexual abstinence. After semen liquefaction, conventional sperm parameters (volume, concentration, and mobility) will be evaluated according to World Health Organization guidelines [70]. Sperm DNA integrity will be assessed with the terminal deoxynucleotidyl transferase dUTP nick-end labelling assay [12] and flow cytometry.

For the members of couples who agreed to receive the intervention only, a detailed multidimensional evaluation will be performed to determine the content of the tailored intervention. Three caregivers, i.e., a sports physician, a dietician, and a psychiatrist, will see in turn and separately each member of the couple during the same half-day, with procedures as follows:

- The sports physician will perform an interrogation (e.g., a familial history of obesity, diabetes, or cardiovascular diseases and personal history of metabolic, endocrinologic, and cardiovascular diseases); a physical examination (i.e., blood pressure, heart rate, BMI, and waist and hip circumferences); a cardiovascular (rest electrocardiogram), rheumatologic, and endocrinologic examination if needed; and a body composition assessment (single-frequency bioelectrical impedance analysis at 50 kHz using the Tanita BC 420 S MA from Tanita Corp., Tokyo, Japan) [71]. The physician also will evaluate physical activity levels through the self-assessed IPAQ questionnaire filled upstream on the web platform.
- The dietician will evaluate diet and eating habits, individual tastes, and abilities. Interrogation is partly based on the results of the Binge eating Scale and a self-reported food frequency questionnaire filled on the web platform. The dietician verifies the responses to the food frequency questionnaire with the patients (e.g., for declared quantities).
- The psychiatrist will screen the participants for depression and anxiety using the self-reported HAD questionnaire. Both members of the couple will undergo a structured clinical interview based on (1) the Miniature International Neuropsychiatric Interview (MINI 5.0) [72] for the diagnosis of psychiatric conditions according to the *Diagnostic and Statistical Manual of Mental Disorders*, fourth edition and (2) on the Alcohol, Smoking and Substance Involvement Screening Test for the screening of substance use disorders.

#### Data collection and assays 3 months after M0 in the intervention group (M3)

Couples included and who have consented to the initial PEPCI intervention (experimental arm) will complete a second consultation with the same sports physician, dietician, and psychiatrist, undergoing the same evaluations as included in M0 (i.e., questionnaires and sample collections).

#### Data collection and assays 12 months after the inclusion or when pregnancy is diagnosed (M12)

Both arms (experimental and control) will be assessed at 12 months from inclusion or sooner in case of pregnancy (when clinical pregnancy is diagnosed, after 6 weeks of amenorrhea) for the following reasons:

- Evaluation of the periconceptional environment through the same self-questionnaires, blood and sperm collection, and anthropometric parameters (biomarkers and biobank) as described above
- Collection of interventions in ART

#### Data collection in the case of pregnancy

The centre coordinators (a physician specialist in reproductive biology and an endocrinologist) will collect data concerning the evolution of the pregnancy until birth.

### Interventions

#### Lifestyle interventions (Fig. 4)

Couples included in the control group will benefit from usual care after the M0 visit. A booklet about a French national nutrition and health program (Programme National Nutrition Santé; PNNS) dedicated to pregnancy will be given to the couple.

**Fig. 4:**
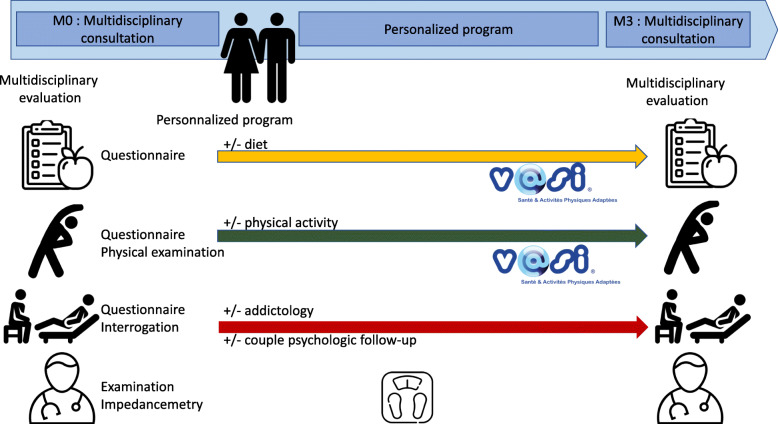
PEPCI intervention program: Experimental group

For the couples accepting the PEPCI intervention (experimental group), a multidisciplinary staff meeting will be organised at the end of the M0 visit. The dietician, endocrinologist, sports physician, psychiatrist, and physician specialist in ART will, together, establish a baseline periconceptional profile from all the M0 assessments to determine two to three individual objectives to be negotiated with both members of the couple. Indeed, the key point is to allocate specific and relevant proposals to each patient from among the following:

1. Diet: If required, a personalised diet prescription according to the personal assessments will be designed with personalised follow-up conducted during the following 3 months through a web platform. Overweight or obese patients will be suggested to enter into a three-month supervised program through a web platform with the possibility to ‘virtually consult’ the dietician up to once a week. The French dietary guidelines (PNNS) can be adapted according to the tastes of the patient, with the a priori objective to lose 1 kg per month [73]. Excessive weight loss in a short period of time has been reported to have a negative effect on the outcome of ART [74] and to be associated with an increased risk of adverse pregnancy outcomes such as low birth weight or miscarriage [75]. In addition, qualitative changes in diet are suggested independently of BMI status. According to the PNNS program, a healthy diet is recommended but the dietician can recommend the patient to change their diet to a Mediterranean one [30, 76]. Finally, the prescription of folic acid (0.4 mg a day) will be systematically checked.
2. Physical activity: A personalised physical activity program individualised to the patients’ physical fitness, preferences, and possibilities will be proposed. Exercise sessions are scheduled online with a certified adapted physical activity trainer via the web platform for 30 min, twice a week. Patient fitness data will be assessed online at M0 and M3 by the trainer, including aerobic fitness with the two-minute step test, a perceived exertion rating using the modified Borg scale [77], submaximal isometric femoral quadriceps strength with the Killy test, abdominal endurance with the curl-up test [78], flexibility of the whole spine and pelvis with the fingertip-to-floor test [79], and unipodal balance with eyes open and closed with the Flamingo test [80]. Adherence to planned sessions will be assessed via the web platform.
3. Psychologic care and addiction: A follow-up with the psychologist will be systematically offered for couples or individuals depending on the wishes of the participants and their needs as assessed by the psychiatrist. The follow-up will consist of three to eight sessions (duration of 45 min) over the 3 months of the intervention. In cases of a psychiatric diagnosis requiring specialised care, patients will be referred to a psychiatrist and not included in the study. If required, a follow-up by a nurse specialised in addiction treatment will be offered. The follow-up consists of three to five sessions (duration of 30 min) of phone counselling over the 3 months of the intervention.

#### ART interventions

All couples, either from the experimental arm or from the control arm, will benefit from a personalised follow-up in the context of usual fertility care, which includes:

- A consultation with a physician specialist in reproductive biology who programs a standardised clinical and biological evaluation of infertility causes
- A multidisciplinary clinical and laboratory staff meeting leading to a decision of a therapeutic strategy, e.g., ovarian stimulation, intrauterine insemination (IUI), in vitro fertilisation (IVF)/intracytoplasmic sperm injection (ICSI).

This usual routine program actually will take at least 3 months and the first ART attempt will occur between three and 12 months since the initial visit (M0).

### Sample size and statistical analysis

We have considered (1) that the PEPCI intervention would lead to a relative risk of clinical pregnancy of 1.6 [28] as compared with usual care (i.e., from 20 to 32% in absolute values, respectively) and (2) that refusal of PEPCI experimental care would intervene for 20% of subjects. The resulting difference, according to the intention-to-treat principle, would therefore be 20% versus 29.6%. Given a two-sided alpha error of 5%, a power of 80%, and a randomisation ratio of 2:1 (control group:experimental group), 466 couples receiving usual care (control group) and 233 couples in the experimental group are required. The total sample size was set to 750 couples (500 control group and 250 experimental group participants), allowing room for a 5% dropout rate.

A flowchart will be used to depict the total participants of each cohort, those per group, and those per gender. All eligible participants will be included in the analyses (since they belong to the global observational cohort), and groups—that is, the randomised control group versus experimental group, encompassing couples having accepted PEPCI and couples having refused PEPCI—will be compared according to the intention-to-treat principle. General and baseline characteristics will be compared between the groups.

An analysis of the primary objective (i.e., the effect of intervention on clinical pregnancy) will be performed with a logistic regression mixed model, with a fixed factor corresponding to the randomisation-arm intervention group and the centre being considered as a random factor. The intervention group will be considered as superior if the lower-limit 95% CI of the corresponding OR of pregnancy is greater than 1. In addition, as a non-negligible proportion of couples may refuse to receive the intervention being trialled, the intention-to-treat analysis may, therefore, dilute any treatment effects. Thus, we will use the statistical method complier average causal-effect analysis, which provides unbiased estimates of the treatment effect for patients who comply with the protocol. This will be considered as a secondary analysis.

The analysis of the effect of the intervention on other criteria (e.g., adherence, satisfaction, BMI and diet, physical activity level, quality of oocytes and semen, fertilisation rate, pregnancy, occurrence of complications) will be performed by a generalised linear mixed-regression model; its link function and distribution of errors will be chosen according to the nature of the response.

No interim analysis will be performed.

#### Ethical, regulatory, and data management considerations

The study protocol has received ethical approvals by the CPP (no. P140934 and no. EUDRACT 2016-AO1281–50 on 22 May 2019). The protocol received authorisation from the ANSM (Agence national de sécurité du medicament et des produits de santé) on 11 February 2016.

The study will be performed in compliance with the Declaration of Helsinki and monitored according to the sponsor’s standard operating procedures. Written informed consent will be retrieved from each participant prior to study enrolment. Data will be recorded on a dedicated e-crf (oneline study notebook) (CleanWEB; Télémédecine Technologies, Boulogne-Billancourt, France). The trial was registered at ClinicalTrials.gov (identifier no. NCT02961907) on 11 November 2016.

The level of monitoring to be set up in this research will be of an intermediate level. The following points will be monitored: (1) existence of included patients, (2) signed informed consent form, (3) eligibility criteria, (4) main criteria, (5) secondary criteria, (6) side effects (e.g., serious side effects, tolerance, new facts), (7) compliance with the monitoring schedule, and (8) data monitoring according to the monitoring guide implemented.

## Discussion

It has been hypothesised that the decrease in male and female fertility could be partially due to environmental and lifestyle factors. Indeed, exposure to pollution, radiation, environmental toxicants, and endocrine disrupters is already known to impact fertility in both males and females [81]. Lifestyle factors such as addictions (tobacco, alcohol, drugs), inadequate diet, lack of physical activity, overweight, obesity, metabolic syndrome, inadequate sleep, and heightened stress levels may also compromise male and female fertility [2, 3, 27, 30, 31, 81]. These lifestyle factors are of interest since they may be reversible and there is a possibility of acting on them.

The PEPCI study has several assets. First, it boasts a holistic approach considering not only inappropriate dietary habits and physical inactivity but also psychological stress, anxiety, and addiction. The intervention is integrated in a global and personalised manner. Importantly, members of couples are involved as partners. Second, not only women but also men are considered. Furthermore, as there are multiple interventions, we will not select only patients with overweight or obesity, as usually is done, but instead individuals with a BMI of less than 49 kg/m^2^.

In addition, an analysis of the effect of a three-month intervention on the targeted factors will allow us to show whether the period of planning pregnancy should be used as a ‘window of opportunity’ to change unhealthy behaviours (M3, second evaluation). Also, with the M12 (at 12 months or when pregnancy is diagnosed) evaluation, we will be able to check whether the effects of the three-month intervention persisted or not.

Fourth, our primary criterion is the clinical ongoing pregnancy rate (i.e. ultrasound determination at 6 weeks of amenorrhea) and not intermediate criteria (according to the pragmatic approach). Usually, ART begins at 3 months after the initial visit and the first 3 months are spent undergoing fertility evaluations. As multidisciplinary interventions are planned during the first 3 months, couples in the intervention group won’t face any additional delay before receiving a first attempt of ART. This was an issue in the study by Mutsaert et al. because women assigned to the lifestyle intervention had access to infertility treatment 6 months later than those in the control group, which induced a lower rate of pregnancies in the follow-up window [19].

Fifth, this controlled trial was conceived as a pragmatic way to investigate the effectiveness of a complex medical intervention under usual conditions. For this purpose, we used the ‘cohort multiple randomised controlled trial’ methodology, with prerandomisation, as accepted by the National Health Service research ethics committee [57] and the French ethics committee to strengthen the external validity by limiting selection bias and recruitment difficulties that may result from initial consent to randomisation. Moreover, for pragmatic trials with a usual care comparator that is available outside the trial, the only incentive to participate is to receive the new intervention, so patients allocated to treatment as usual may be disappointed and may withdraw from the trial (attrition bias). Lastly, all eligible people are followed within the cohort to obtain realistic estimates of adherence, benefits and adverse events, if any exist. One of the limitations of this study is that full blinding will not be possible; however, the main criteria will be assessed blindly. With this methodology, blinding for patients in the control group will be preserved.

The weakness of this study is the absence of a pilot study that would anticipate its feasibility. In addition, there is no conceptual model of the intervention established, although we determined the nutritional program to be applied according to the available literature at the time of study conception. Furthermore, for economic reasons, we had to opt for a 2:1 design. There are also limitations with deploying an intervention via an Internet platform since there are equipment constraints. Finally, the multidisciplinary approach and the possibility for the patient to choose the intervention allows for tailor-made patient care but it makes it difficult to explain the factors that might have actually influenced the birth.

### Perspectives

The PEPCI results might show that an early diagnosis of unhealthy habits and adherence to the recommendations of physicians and follow-up are be associated with an increased chance of ongoing pregnancy and live birth spontaneously or after the first ART treatment (IUI, IVF or ICSI).

This may also be a crucial issue considering the developmental origin of health and disease (DOHaD). Indeed, current evidence indicates that unhealthy inappropriate preconceptional diet and lifestyle in both men and women not only contributes to impaired reproduction with potential long-term consequences for parental health but also compromises the health of their offspring [82, 83].

To conclude, the proposed PEPCI project might provide evidence that programs, aimed at beneficially changing preconception nutritional and lifestyle factors, should be considered as a first-choice treatment for unexplained infertility and should be suggested to each couple before ART. This original research program could also lead to public recommendations of periconceptional habits not only for subfertile couples but for all parents-to-be.

## Data Availability

Data are the property of the Public Assistance – Paris Hospitals [Assistance Publique – Hôpitaux de Paris (AP-HP)] that does not authorize as a promoter the sharing of data without a contract. Consultation by the editorial board or interested researchers may nevertheless be considered.

## Abbreviations

ART: Assisted reproductive technologies
BMI: Body mass index
DNA: Deoxyribonucleic acid
DOHAD: Developmental origin of health and disease
ECG: Electrocardiogram
FIV: In vitro fertilization
HAD: Hospital Anxiety and Depression scale
HDL: High-density lipoprotein cholesterol
ICSI: Intracytoplasmic sperm injection
IIU: Intrauterine insemination
IPAQ: International Physical activity Questionnaire
LDL: Low-density lipoprotein cholesterol
M0: Baseline
M12: 12 months later
M3: 3 months later
PEPCI: Parcours environnement périconceptionnel en infertilité
PNNS: Programme national nutrition santé
PSQI: Pittsburgh sleep quality index
RR: Risk ratio
SUVIMAX: SUpplémentation en vitamines et minéraux antioxydants
TUNEL: Terminal deoxynucleotidyl transferase dUTP nick-end labeling
